# Bis(2-amino-4-methyl­pyridinium) tetra­chloridocuprate(II)

**DOI:** 10.1107/S1600536808041652

**Published:** 2008-12-17

**Authors:** Rawhi H. Al-Far, Basem Fares Ali

**Affiliations:** a Faculty of Information Technology and Science, Al-Balqa’a Applied University, Salt, Jordan; b Department of Chemistry, Al al-Bayt University, Mafraq 25113, Jordan

## Abstract

The asymmetric unit of the title compound, (C_6_H_9_N_2_)_2_[CuCl_4_], consists of one cation and one half-anion, bis­ected by a twofold rotation axis through the metal center. The anion exhibits a geometry that is inter­mediate between a *T_d_* and *D*_4*h*_ arrangement about the Cu atom. The crystal structure contains chains of cations alternating with stacks of anions. The cationic groups inter­act *via* offset face-to-face π–π stacking, forming chains running along the *c* axis. The anion stacks are parallel to the cation chains, with no significant inter- nor intra­stack Cl⋯Cl inter­actions. There are several anion–cation hydrogen-bonding inter­actions of the (N—H)_pyridine_⋯Cl and (N—H)_amino_⋯Cl types, connecting the chains of cations to the stacks of anions. Both the N—H⋯Cl and π–π stacking inter­actions [centroid–centroid distances 3.61 (8) and 3.92 (2) Å] contribute to the formation of a three-dimensional supra­molecular architecture.

## Related literature

For related literature on organic–inorganic hybrids, see: Al-Far, Ali & Haddad (2008 ▶); Ali & Al-Far (2007 ▶, 2008 ▶); Coffey *et al.* (2000 ▶). For bond-length and angle data, see: Raithby *et al.* (2000 ▶); Allen *et al.* (1987 ▶).
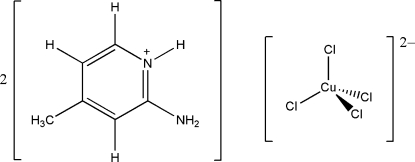


## Experimental

### 

#### Crystal data

(C_6_H_9_N_2_)_2_[CuCl_4_]
*M* *_r_* = 423.65
Monoclinic, 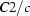

*a* = 11.313 (3) Å
*b* = 12.272 (3) Å
*c* = 14.264 (4) Å
β = 113.201 (17)°
*V* = 1820.2 (9) Å^3^
*Z* = 4
Mo *K*α radiation
μ = 1.78 mm^−1^
*T* = 293 (2) K
0.35 × 0.06 × 0.06 mm

#### Data collection

Siemens P4 diffractometer
Absorption correction: multi-scan (*SADABS*; Bruker, 2005 ▶) *T*_min_ = 0.874, *T*_max_ = 0.898
2039 measured reflections
1590 independent reflections
841 reflections with *I* > 2σ(*I*)
*R*_int_ = 0.058
3 standard reflections every 97 reflections intensity decay: none

#### Refinement

*R*[*F* ^2^ > 2σ(*F* ^2^)] = 0.059
*wR*(*F* ^2^) = 0.145
*S* = 0.99
1590 reflections
97 parameters
H-atom parameters constrained
Δρ_max_ = 0.40 e Å^−3^
Δρ_min_ = −0.37 e Å^−3^

### 

Data collection: *XSCANS* (Bruker, 1996 ▶); cell refinement: *XSCANS*; data reduction: *SHELXTL* (Sheldrick, 2008 ▶); program(s) used to solve structure: *SHELXS97* (Sheldrick, 2008 ▶); program(s) used to refine structure: *SHELXL97* (Sheldrick, 2008 ▶); molecular graphics: *SHELXTL*; software used to prepare material for publication: *SHELXTL*.

## Supplementary Material

Crystal structure: contains datablocks I, New_Global_Publ_Block. DOI: 10.1107/S1600536808041652/bg2228sup1.cif

Structure factors: contains datablocks I. DOI: 10.1107/S1600536808041652/bg2228Isup2.hkl

Additional supplementary materials:  crystallographic information; 3D view; checkCIF report

## Figures and Tables

**Table:** 

Cu1—Cl1	2.2614 (19)
Cu1—Cl2	2.2698 (19)

**Table:** 

Cl1^i^—Cu1—Cl1	94.33 (10)
Cl1—Cu1—Cl2	146.17 (8)

Symmetry code: (i) 
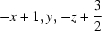
.

**Table 2:** Hydrogen-bond geometry (Å, °)

*D*—H⋯*A*	*D*—H	H⋯*A*	*D*⋯*A*	*D*—H⋯*A*
N1—H1*A*⋯Cl1	0.86	2.65	3.407 (6)	147
N1—H1*A*⋯Cl2^i^	0.86	2.70	3.360 (6)	134
N2—H2*A*⋯Cl1	0.86	2.50	3.294 (6)	153
N2—H2*B*⋯Cl2^ii^	0.86	2.53	3.359 (6)	164

Symmetry codes: (i) 
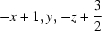
; (ii) 
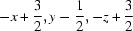
.

**Fig. 1.**
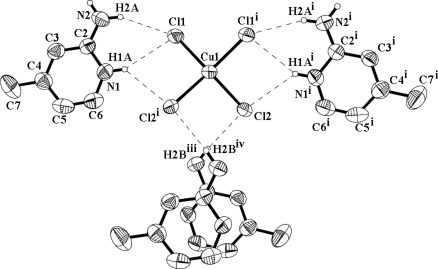
The structure of the title compound, viewed down c. Displacement ellipsoids are drawn at the 50% probability level. N—H···Cl—Cu intermolecular interactions are shown as dashed lines. Symmetry operations: (i) -x + 1, y, -z + 3/2; (iii) -1/2 + x, 1/2 + y, z; (iv) 3/2 - x, 1/2 + y, 3/2 - z. H atoms not involved in hydrogen bonding omitted for clarity.

**Fig. 2.**
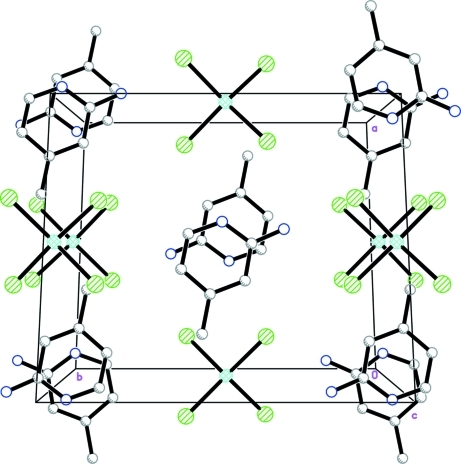
Crystal packing diagram showing alternating stacks of anions and chains of cations.

**Fig. 3.**
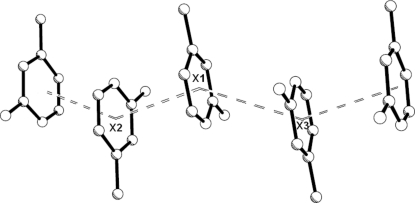
Cationic chains along the crystallographic c axis, assembled via offset face-to-face (π–π stacking; double broken lines) motifs. Centroids separation distances are X2(2 - x, y, 3/2 - z)···X1 (x, y, z)···X3(2 - x, - y, 2 - z) are 3.61 (8) and 3.92 (2) Å, respectively.

## supplementary crystallographic information

### Comment

Hybrid organic-inorganic low dimensional magnetic lattices of the formula (cation)_2_[*MX*_4_] are of special interest (Coffey *et al.*, 2000; and references therein). A wide variety of these complexes are known. Some examples are those containing a protonated pyridine and 2-aminopyrimidine (Coffey *et al.*, 2000). The magnetic exchange in these compounds is mediated by van der Waals contacts between the halide ions of the [*MX*_4_]^2-^ pseudo-tetrahedra and the contacts are determined by the crystal packing. In connection with ongoing studies (Al-Far *et al.*, 2008; Ali & Al-Far 2008; Ali & Al-Far 2007) of the structural aspects of organic-inorganic hybrids, here we report the crystal structure of Cu(II)-chloride complex with 2-amino-4-methylpyridinium as the organic cation.

The asymmetric unit in I contains one half anion (bisected by a two fold axis through the metal) and one cation (Fig. 1). The Cu—Cl distances and Cl—Cu—Cl, angles, Table 1,  fall in the range reported previously for compounds containing Cu—Cl anions (Raithby *et al.*, 2000). The CuCl_4_^2-^ anion geometry is an intermediate between regular tetrahedral (*T_d_*) and square planar (*D_4_**_h_*); the geometry of Cu*X*_4_^2-^ anions will always distort from *T_d_* due to the Jahn-Teller effect, and this generally results in a compressed tetrahedral geometry. The extent of this compression is determined principally by electrostatic interactions with the environment – in this case, the hydrogen bonding.

In the cation bond lengths and angles are in accordance with normal values (Allen *et. al.*, 1987).

The crystal packing (Fig. 2) show alternating stacks of anions and chains of cations. The anion stacks are parallel to the cation chains, with no significant inter- and intra-stack Cl···Cl interactions. The cations interact *via* offset face-to-face, π–π stacking interactions leading to chains along the crystallographic *c* axis (Fig. 3), with alternating rings centroids separation distances of 3.61 (8) and 3.92 (2) Å.

There are extensive cation···anion intermolecular interactions (Table 2; Fig. 1). In these interactions H1A is involved in a bifurcated hydrogen bonding motif with Cl and Cl2^i^ [N1—H1A···(Cl1,Cl2^i^) distances are 3.407 (6) and 3.360 (6) Å, respectively, with N1—H1A···(Cl1,Cl2^i^) angles being 147 and 134°; Symmetry codes: (i) -*x* + 1, *y*, -*z* + 3/2]. The other interactions result between N2—H2A···Cl1 [N1···Cl1 distance is 3.294 (6) and N2—H2A···Cl1 angle of 153°] and N2—H2B···Cl2^ii ^[with N2···Cl2^ii ^distance of 3.359 (6) Å and N2—H2B···Cl2^ii ^angle being 164°; Symmetry code: (ii) -*x* + 3/2, *y* - 1/2, -*z* + 3/2]. These interactions and the symmetrically related ones connect the anion to four surrounding cations.

Both N—H···Cl and π–π stacking interactions cause to the formation of a three-dimensional supramolecular architecture.

### Experimental

To a hot solution (100 °C) of 2-Amino-4-methylpyridine (1 mmol) in 5 ml of CH_3_CN acidified with 2 ml of 3 *M* HCl, CuCl_2_.2H_2_O (1 mmol) dissolved in 10 ml CH_3_CN was added. The resulting mixture was refluxed for 1.5 h. The solution was then allowed to stand undisturbed at room temperature. After 24 h yellow parallelepiped crystals were formed (yield: 0.170 g; 80.2%).

### Refinement

Hydrogen atoms were positioned geometrically, with N—H = 0.86 Å, C—H = 0.93 Å for aromatic H and C—H = 0.96 Å for methyl H, and constrained to ride on their parent atoms, *U*_iso_(H) = *xU*_eq_(*C,N*), where *x* = 1.5 for methyl H, and *x* = 1.2 for all other H atoms.

### Crystal data

**Table:**

(C_6_H_9_N_2_)_2_[CuCl_4_]	*F*(000) = 860
*M**_r_* = 423.65	*D*_x_ = 1.546 Mg m^−^^3^
Monoclinic, *C*2/*c*	Mo *K*α radiation, λ = 0.71073 Å
Hall symbol: -C 2yc	Cell parameters from 290 reflections
*a* = 11.313 (3) Å	θ = 2.5–27.3°
*b* = 12.272 (3) Å	µ = 1.78 mm^−^^1^
*c* = 14.264 (4) Å	*T* = 293 K
β = 113.201 (17)°	Parallelepiped, yellow
*V* = 1820.2 (9) Å^3^	0.35 × 0.06 × 0.06 mm
*Z* = 4	

### Data collection

**Table:**

Siemens P4 diffractometer	841 reflections with *I* > 2σ(*I*)
Radiation source: fine-focus sealed tube	*R*_int_ = 0.058
graphite	θ_max_ = 25.0°, θ_min_ = 2.6°
ω scans	*h* = −1→13
Absorption correction: multi-scan (*SADABS*; Bruker, 2005)	*k* = −14→1
*T*_min_ = 0.874, *T*_max_ = 0.898	*l* = −16→16
2039 measured reflections	3 standard reflections every 97 reflections
1590 independent reflections	intensity decay: none

### Refinement

**Table:**

Refinement on *F*^2^	Primary atom site location: structure-invariant direct methods
Least-squares matrix: full	Secondary atom site location: difference Fourier map
*R*[*F*^2^ > 2σ(*F*^2^)] = 0.059	Hydrogen site location: inferred from neighbouring sites
*wR*(*F*^2^) = 0.145	H-atom parameters constrained
*S* = 0.99	*w* = 1/[σ^2^(*F*_o_^2^) + (0.0577*P*)^2^] where *P* = (*F*_o_^2^ + 2*F*_c_^2^)/3
1590 reflections	(Δ/σ)_max_ < 0.001
97 parameters	Δρ_max_ = 0.40 e Å^−^^3^
0 restraints	Δρ_min_ = −0.37 e Å^−^^3^

### Special details

**Table:**

Geometry. All e.s.d.'s (except the e.s.d. in the dihedral angle between two l.s. planes) are estimated using the full covariance matrix. The cell e.s.d.'s are taken into account individually in the estimation of e.s.d.'s in distances, angles and torsion angles; correlations between e.s.d.'s in cell parameters are only used when they are defined by crystal symmetry. An approximate (isotropic) treatment of cell e.s.d.'s is used for estimating e.s.d.'s involving l.s. planes.
Refinement. Refinement of *F*^2^ against ALL reflections. The weighted *R*-factor *wR* and goodness of fit *S* are based on *F*^2^, conventional *R*-factors *R* are based on *F*, with *F* set to zero for negative *F*^2^. The threshold expression of *F*^2^ > σ(*F*^2^) is used only for calculating *R*-factors(gt) *etc*. and is not relevant to the choice of reflections for refinement. *R*-factors based on *F*^2^ are statistically about twice as large as those based on *F*, and *R*- factors based on ALL data will be even larger.

### Fractional atomic coordinates and isotropic or equivalent isotropic displacement parameters (Å^2^)

**Table:**

	*x*	*y*	*z*	*U*_iso_*/*U*_eq_	
Cu1	0.5000	0.00098 (10)	0.7500	0.0539 (4)	
Cl1	0.64416 (17)	−0.12430 (14)	0.74530 (17)	0.0716 (7)	
N1	0.9282 (5)	0.0108 (5)	0.8601 (4)	0.0552 (15)	
H1A	0.8473	0.0000	0.8432	0.066*	
Cl2	0.35031 (15)	0.12618 (14)	0.66064 (15)	0.0632 (6)	
C2	1.0055 (6)	−0.0772 (6)	0.8719 (5)	0.0487 (18)	
N2	0.9548 (6)	−0.1746 (5)	0.8554 (5)	0.0736 (19)	
H2A	0.8732	−0.1825	0.8372	0.088*	
H2B	1.0030	−0.2309	0.8626	0.088*	
C3	1.1373 (6)	−0.0577 (6)	0.9018 (5)	0.0551 (19)	
H3A	1.1933	−0.1161	0.9116	0.066*	
C4	1.1850 (7)	0.0474 (7)	0.9168 (5)	0.0584 (19)	
C5	1.0969 (9)	0.1339 (6)	0.9006 (6)	0.072 (2)	
H5A	1.1258	0.2057	0.9089	0.087*	
C6	0.9713 (9)	0.1124 (6)	0.8733 (6)	0.071 (2)	
H6A	0.9138	0.1697	0.8636	0.085*	
C7	1.3250 (7)	0.0704 (8)	0.9462 (7)	0.095 (3)	
H7A	1.3691	0.0038	0.9454	0.143*	
H7B	1.3604	0.1013	1.0134	0.143*	
H7C	1.3352	0.1209	0.8985	0.143*	

### Atomic displacement parameters (Å^2^)

**Table:**

	*U*^11^	*U*^22^	*U*^33^	*U*^12^	*U*^13^	*U*^23^
Cu1	0.0460 (7)	0.0348 (6)	0.0783 (9)	0.000	0.0215 (6)	0.000
Cl1	0.0511 (11)	0.0382 (10)	0.1332 (19)	−0.0006 (8)	0.0444 (11)	−0.0103 (11)
N1	0.049 (3)	0.052 (4)	0.064 (4)	0.006 (3)	0.022 (3)	−0.005 (3)
Cl2	0.0447 (10)	0.0374 (10)	0.0867 (14)	−0.0050 (8)	0.0037 (8)	0.0074 (9)
C2	0.050 (4)	0.046 (4)	0.049 (4)	0.004 (4)	0.020 (3)	0.000 (3)
N2	0.059 (4)	0.043 (4)	0.121 (6)	−0.008 (3)	0.038 (4)	−0.007 (4)
C3	0.057 (5)	0.049 (5)	0.062 (5)	0.007 (4)	0.027 (4)	−0.002 (4)
C4	0.063 (5)	0.058 (5)	0.055 (5)	−0.007 (4)	0.023 (4)	0.002 (4)
C5	0.097 (7)	0.041 (5)	0.080 (6)	−0.006 (5)	0.035 (5)	−0.003 (4)
C6	0.073 (6)	0.049 (5)	0.085 (6)	0.008 (5)	0.026 (5)	−0.004 (5)
C7	0.078 (6)	0.104 (8)	0.109 (7)	−0.038 (6)	0.044 (6)	−0.031 (6)

### Geometric parameters (Å, °)

**Table:**

Cu1—Cl1^i^	2.2615 (19)	C3—C4	1.381 (9)
Cu1—Cl1	2.2614 (19)	C3—H3A	0.9300
Cu1—Cl2^i^	2.2698 (19)	C4—C5	1.412 (10)
Cu1—Cl2	2.2698 (19)	C4—C7	1.496 (10)
N1—C6	1.326 (9)	C5—C6	1.343 (11)
N1—C2	1.358 (8)	C5—H5A	0.9300
N1—H1A	0.8600	C6—H6A	0.9300
C2—N2	1.307 (8)	C7—H7A	0.9600
C2—C3	1.400 (9)	C7—H7B	0.9600
N2—H2A	0.8600	C7—H7C	0.9600
N2—H2B	0.8600		
			
Cl1^i^—Cu1—Cl1	94.33 (10)	C2—C3—H3A	119.6
Cl1^i^—Cu1—Cl2^i^	146.17 (8)	C3—C4—C5	118.0 (7)
Cl1—Cu1—Cl2^i^	95.15 (7)	C3—C4—C7	121.7 (7)
Cl1^i^—Cu1—Cl2	95.15 (6)	C5—C4—C7	120.3 (8)
Cl1—Cu1—Cl2	146.17 (8)	C6—C5—C4	119.8 (8)
Cl2^i^—Cu1—Cl2	94.80 (10)	C6—C5—H5A	120.1
C6—N1—C2	123.2 (7)	C4—C5—H5A	120.1
C6—N1—H1A	118.4	N1—C6—C5	120.9 (8)
C2—N1—H1A	118.4	N1—C6—H6A	119.5
N2—C2—N1	119.3 (6)	C5—C6—H6A	119.5
N2—C2—C3	123.4 (7)	C4—C7—H7A	109.5
N1—C2—C3	117.3 (7)	C4—C7—H7B	109.5
C2—N2—H2A	120.0	H7A—C7—H7B	109.5
C2—N2—H2B	120.0	C4—C7—H7C	109.5
H2A—N2—H2B	120.0	H7A—C7—H7C	109.5
C4—C3—C2	120.8 (7)	H7B—C7—H7C	109.5
C4—C3—H3A	119.6		
			
C6—N1—C2—N2	−178.6 (7)	C2—C3—C4—C7	−178.4 (7)
C6—N1—C2—C3	1.5 (10)	C3—C4—C5—C6	1.2 (11)
N2—C2—C3—C4	179.1 (7)	C7—C4—C5—C6	179.3 (8)
N1—C2—C3—C4	−1.0 (10)	C2—N1—C6—C5	−0.6 (12)
C2—C3—C4—C5	−0.3 (11)	C4—C5—C6—N1	−0.8 (12)

Symmetry codes: (i) −*x*+1, *y*, −*z*+3/2.

### Hydrogen-bond geometry (Å, °)

**Table:**

*D*—H···*A*	*D*—H	H···*A*	*D*···*A*	*D*—H···*A*
N1—H1A···Cl1	0.86	2.65	3.407 (6)	147
N1—H1A···Cl2^i^	0.86	2.70	3.360 (6)	134
N2—H2A···Cl1	0.86	2.50	3.294 (6)	153
N2—H2B···Cl2^ii^	0.86	2.53	3.359 (6)	164

Symmetry codes: (i) −*x*+1, *y*, −*z*+3/2; (ii) −*x*+3/2, *y*−1/2, −*z*+3/2.
